# Antihypertensive treatments for spontaneous intracerebral hemorrhage in patients with cerebrovascular stenosis: A randomized clinical trial (ATICHST)

**DOI:** 10.1097/MD.0000000000007289

**Published:** 2017-06-30

**Authors:** Zengpanpan Ye, Xiaolin Ai, Jun Zheng, Xin Hu, Sen Lin, Chao You, Hao Li

**Affiliations:** Department of Neurosurgery, West China Hospital, Sichuan University, Chengdu, Sichuan, China.

**Keywords:** antihypertensive treatment, cerebral blood flow, cerebral ischemia, stenosis

## Abstract

**Introduction:**

Antihypertensive treatment is associated with clinical outcomes in patients with spontaneous intracerebral hemorrhage (sICH). ADAPT showed that intensive blood pressure lowering (<140 mm Hg) does not reduce peri-hematoma regional cerebral blood flow (rCBF) in patients with sICH. However, the stenosis of main cerebral arteries that has a high presence in patients with sICH is well-known related to the brain ischemia. The effect of intensive BP lowering for sICH in patients with cerebrovascular stenosis is still unknown.

**Aim:**

The aim of this study was to determine the safety and effectiveness of intensive BP lowering for sICH in patients with cerebrovascular stenosis.

**Methods and analysis:**

A pilot trial has been conducted to calculate the sample size and 80 patients of sICH with cerebrovascular stenosis will be involved. The target of systolic blood pressure (SBP) will be maintained at from 120 to 140 mm Hg or from 140 to 180 mm Hg for 7 days. Cerebral ischemia will be assessed at 24 hours after onset by computed tomography (CT) perfusion imaging and the follow-up will be conducted at 30-day and 90-day. The primary outcome is the reduction of peri-hematoma rCBF. The other cerebral perfusion indexes and the rate of ischemic stroke are regarded as other primary outcomes. The secondary outcomes include clinical outcome at 30 days and 90 days, complications, and hospital stays.

**Discussion:**

The ATICHST trial has been signed as a parallel, prospective, randomized, assessor-blinded clinical trial to determine the effects of intensive BP lowering on sICH in patients with cerebrovascular stenosis, the results of which will contribute to guide the management of blood pressure in sICH.

**Conclusion:**

The protocol will determine the safety and effectiveness of intensive BP lowering for sICH with cerebrovascular stenosis.

## 1 Background

Spontaneous intracerebral hemorrhage (sICH) is the second most prevalent cause of stroke, and almost 1 million patients are diagnosed with sICH every year worldwide.^[1,2]^ sICH is associated with a high rate of mortality^[3]^ and disability.^[1,4]^

Recently, the management of blood pressure (BP) has been bone of contention for the sICH patients. Almost 90%^[5,6]^ of acute sICH patients have an elevated systolic BP (SBP) caused by the elevated intracranial pressure to keep cerebral blood +flow (CBF)^[7]^ and the highest SBP always appears at 24 hours after onset.^[8,9]^ The elevated BP increases the risk of hematoma expansion,^[10]^ while the acute lowering of BP may result in the ischemia of brain tissue.^[11]^

Several high-quality, multicentric, randomized controlled trials^[12,13]^ suggested the intensive BP lowering was safety in sICH patients and the ADAPT trial showed the peri-hematoma rCBF did not decrease after intensive BP lowering.^[14]^ A meta-analysis^[15]^ including 1427 patients also suggested that intensive BP lowering was safe and might have a potency of reducing hematoma expansion and improving clinical outcome. However, all of the mentioned randomized controlled trials and related clinical studies have no subgroup to discuss sICH with cerebrovascular stenosis and the management of BP for sICH patient with cerebrovascular stenosis is still unknown.

Brain ischemia has a high incidence of 25%^[16,17]^ in patients with sICH. The hematoma is surrounded by an ischemic penumbra, which needs adequate CBF to maintain its viability.^[18]^ The intensive SBP lowering may aggravate ischemia in ischemic penumbra and results in 20% of sICH patients suffering from the brain ischemia or infarction.^[11]^

The cerebrovascular stenosis has a rate of 20% to 54%^[19]^ in sICH patients, including intracranial cerebrovascular stenosis and extracranial cerebrovascular stenosis.^[20]^ The rate of intracranial cerebrovascular stenosis (16%) was higher than extracranial cerebrovascular stenosis (6%) in Chinese.^[21]^ Many researches^[22–26]^ demonstrated that 18% to 24% of ischemic stroke was closely associated with the stenosis of main cerebral vessels. When cerebrovascular stenosis coexists with sICH, intensive SBP lowering could increase the risk of ischemia and infarction.^[20,27]^

Theoretically, the conservative antihypertensive treatment (<180 mm Hg) is safe and may be beneficial to the sICH patients with cerebrovascular stenosis. The patients undergoing conservative antihypertensive treatment may have higher peri-hematoma rCBF and lower incidence of brain ischemia or infarction, which are associated with neurological deterioration or mortality. Moreover, long-term outcome may be better for the neuroprotection after the conservative antihypertensive treatment. Thus, a well-designed clinical trial is need to assess the safety and efficacy of intensive SBP lowering for sICH with cerebrovascular stenosis, and provides powerful evidence to clinical practice. Here, we described a parallel, superiority, randomized, assessor-blinded trial termed antihypertensive treatment in patients of sICH with cerebrovascular stenosis trial (ATICHST).

## 2 Methods

### 2.1 Design

**Figure 1:**
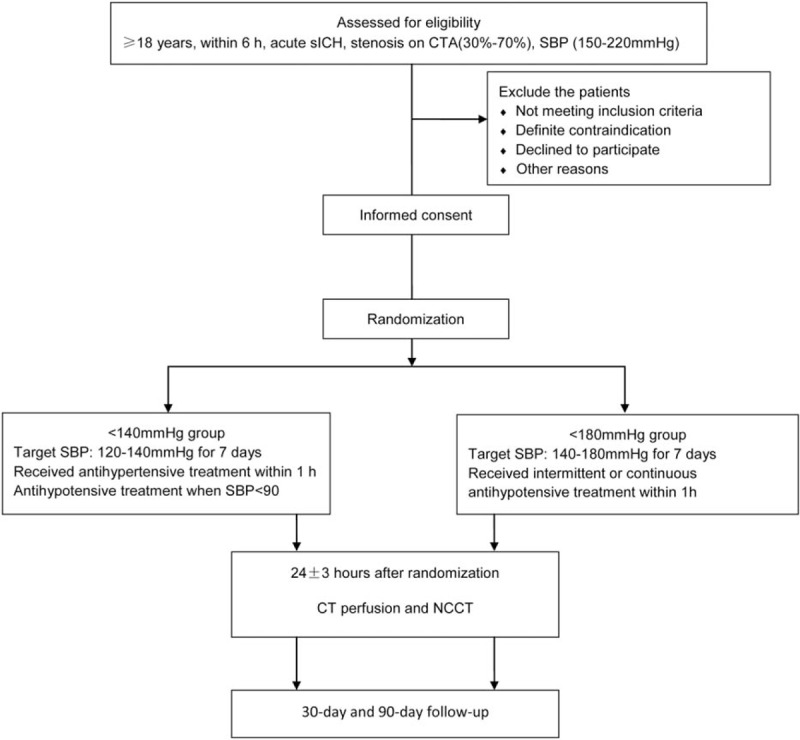
Flow chart of the clinical trial. CTA = CT angiography, NCCT = noncontrast CT, SBP = systolic blood pressure, sICH = spontaneous intracerebral hemorrhage.

The ATICHST is a parallel, prospective, randomized, assessor-blinded clinical trial of intensive BP-lowering treatment and conservative BP-lowering treatment in the sICH patients with cerebrovascular stenosis, which is conducted according to the flow diagram (Fig. 1) by the Department of Neurosurgery in West China Hospital, Sichuan University. The protocol has received approval from the Biological and Medical Ethics Committee of West China Hospital (2016 Reviewed-No. 331). This protocol is also registered in the Chinese Clinical Trial Registry (ChiCTR-IOR-17010675). The written informed consent is obtained from each participant or their legal surrogate after fully informed.

### 2.2 Study objective

The key primary objective is to compare the relative reduction of peri-hematoma rCBF in sICH patients with cerebrovascular stenosis under different BP treatments and discuss whether the degree of stenosis or location of stenosis on relative main arteries has an effect on the peri-hematoma rCBF by analysis of subgroups. The other primary objectives are to analyze the absolute change of cerebral perfusion indexes, the occurrence rate of ischemic stroke, and incidence of hematoma expansion within 7 days. The key second objective is to determine the effects of different BP managements on unfavorable clinical outcome at 90-day in sICH patients with cerebrovascular stenosis. The other secondary objectives are to assess the effects of the treatments separately on death and dependency through physical function, health-related quality of life, other vascular events, and hospital stays.

### 2.3 Patients population

#### 2.3.1 Inclusion criteria

(1) Age ≥18 years;
(2) Randomized to groups within 6 hours from onset;
(3) Acute spontaneous ICH, verified by noncontrast CT (NCCT) or magnetic resonance imaging;
(4) Cerebrovascular luminal narrowing (30–70%) demonstrated with CT angiography (CTA) upon admission;
(5) SBP is from 150 to 220 mm Hg (twice repeat measurements, record ≥2 minutes apart)

#### 2.3.2 Exclusion criteria

(1) Known definite contraindication to intensive BP-lowering treatment (e.g., severe cerebrovascular stenosis (>70%), severe aortic valve stenosis, severe renal failure);
(2) Known definite indication to intensive BP-lowering treatment (e.g., very high SBP ≥220 mm Hg; hypertensive encephalopathy; aortic dissection);
(3) The ICH is caused by secondary factors (e.g., intracranial aneurysm; arteriovenous malformation; tumor stroke; anticoagulant correlation hemorrhage).
(4) Glass coma scale ≤5 or deep coma;
(5) Disabled or with underlying diseases (e.g., known dementia, severe cardiovascular disease, kidney failure) before onset;
(6) History of ischemic stroke within 30 days before onset of sICH;
(7) Known indication of emergency surgery (volume of supratentorial hematoma ≥30 mL or volume of infratentorial hematoma ≥10 mL; within 48 hours from onset; Glass coma scale ≥5);
(8) Contraindication to CT perfusion imaging (CTP) (e.g., contrast allergy, pregnancy, or creatinine ≥160 mmol/L).

### 2.4 Sample size

The initial assessment of sample size is based on taking peri-hematoma rCBF as the ischemic reign of brain tissue.^[14]^ For no study now available referring to the field, the pilot trial was conducted to assess the sample size.

The pilot data includes 12 patients (6/each group) and shows the rate of reduction of peri-hematoma rCBF are (0.4282 ± 0.14705) and (0.3555 ± 0.12592) in intensive/conservative BP-lowering group, compared with that of contralateral homologous reign. For the reduction of relative rCBF, ≥44%^[28]^ is regarded as a threshold of infarction, if the difference of 2 groups is more than 9% (9%+35.5%>44%); acute SBP lowering is considered to significantly increase the risk of infarction in these patients. So, each sample size of 36 in a group will be required to detect a significant difference of 5% (2-tailed) and a power of 90% (PASS software Version 11.0.4; NCSS). In view of 10% patients lost to follow-up, a total of 80 patients (40 in each group) will be included in this trial.

### 2.5 Randomization

Patients are assigned to intensive BP-lowering group and conservative BP-lowering group by a minimization random allocation system (minimpy 0.3). They are stratified by level of stenosis and location of stenosis. All information of patients is collected and randomization is stratified by a special researcher within 1 hour after admission in department of emergency. Once the group is determined, the allocation is not allowed to change unless quit from the trial.

### 2.6 Blinding

Only the assessors are blinded to this trial because BP targets cannot be concealed to the patients and doctors. The Case Reports Forms (CRFs) will be completed by 2 special assessors who are not involved in the design and assignment. There is no information about the assignment on the CRF and level of BP. The patients and doctors will be informed not to leak the level of BP to the assessors. All data of each group will be analyzed by an analyst who is not involved in this trial.

### 2.7 Treatment

After informed fully, participants or their legal surrogate write informed consent. Participants are allocated into intensive group or conservative group after randomization.

#### 2.7.1 Intensive group

All patients receive a standardized, stepped, continuous intravenous infusion of antihypertensive agents and frequent BP monitoring. On the basis of the 2015 guidelines of American Heart Association (AHA),^[29]^ the SBP intensive group is to maintain below 140 mm Hg within 1 hour from randomization. The intravenous antihypertensive agents included nitroglycerin, nicardipine, and urapidil hydrochloride. With the use of oral antihypertensive agents after the safety evaluation by doctor group, the doses of intravenous antihypertensive agents are reduced gradually. SBP is maintained from 120 to 140 mm Hg for following 7 days or until hospital discharge if this occurred in advance. About 120 mm Hg is considered as the safe threshold value, lower than which patients may have a risk of low BP. When SBP is lower than 120 mm Hg, we will reduce the velocity of infusion and adjust it according to repeated measurements of BP until SBP maintaining 120 to 140 mm Hg. All of antihypertensive agents are stopped when SBP is lower than 90 mm Hg, and antihypotensive treatments will initiate if necessary. The oral antihypertensive agents are suggested to use to maintain the BP <140 mm Hg upon discharge.

#### 2.7.2 Conservative group

The goal of conservative group is to maintain SBP from 140 to 180 mm Hg within 1 hour after randomization. The intermittent or continuous intravenous antihypertensive agents are used to maintain the SBP from 140 to 180 mm Hg and be examined again every 15 minutes. When SBP is more than 180 mm Hg, we will increase the dose of antihypertensive agents or combine different antihypertensive agents.

#### 2.7.3 Imaging procedures

A standard NCCT and CTA are conducted when admitted in emergency department, by a 64-slice CT scanner (SOMATOM Definition Flash; Siemens Healthcare Sector, Forchheim, Germany). The scanning parameters of NCCT are 120 kVp, 340 mA, and 5 mm slice thickness. The hematoma volume is calculated by the ABC/2 method.^[30]^ CTA is initiated after the infusion of 100-mL contrast material (80 kVp, 110 mA, 1 mm slice thickness, and pitch 1:1). 3D reconstructions of CTA are performed to assess the level of cerebrovascular stenosis. NCCT (120 kVp, 340 mA, and 5 mm slice thickness) and CTP are conducted 24 ± 3 hours after randomization in order to assess for additional HE and perihaematoma rCBF with different treatments. The CTP will be initialed after the infusion of 42 mL contrast material given over 12 seconds with CT images acquired every 1.5 seconds at most 50 seconds (70 kVp, 150 mA, 1 mm slice thickness).

#### 2.7.4 Primary outcomes

The relative reduction of the perihematoma rCBF is considered as the key primary outcome. Relative reduction of rCBF = 1-(rCBF / contralateral homologous regions rCBF).^[14]^ The other primary outcomes include absolute CBF, Tmax, mean transit time (MTT), and rCBV of perihematoma region, those indexes of global and hemisphere, the rate of ischemic stroke within 7 days, volumes of parenchymal hematoma, IVH, and ischemic region.

#### 2.7.5 Image analysis: CTA and CT perfusion

**Figure 2:**
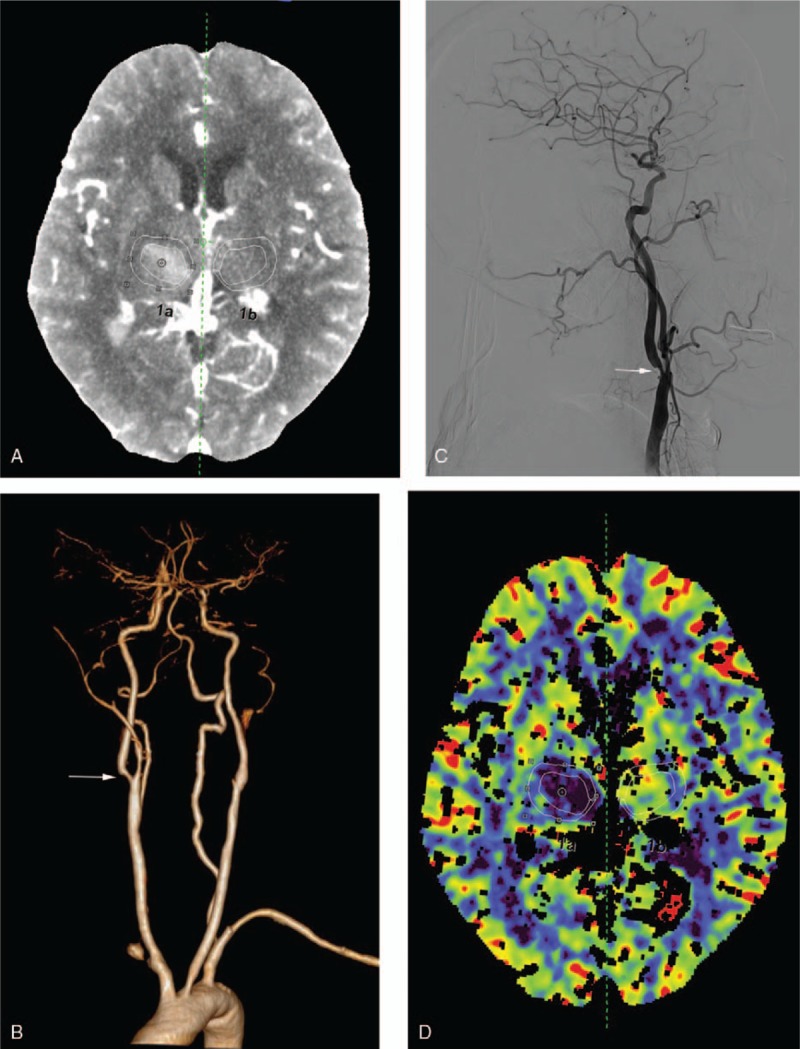
A 58-year-old female was diagnosed with right basal ganglion hemorrhage by NCCT (A). CTA (B) and DSA (C) identified the 62% stenosis of right internal carotid artery with white arrow. The CTP showed the mean CBF of perihematoma (20.24 mL/100 mL/min) and contralateral homologous region (48.05 mL/100 mL/min).

The stenosis is defined as the reduction of an artery diameter ≥50%^[31–33]^ or occlusion with digital subtraction angiography (DSA), magnetic resonance angiography, or CTA, and severe is defined as ≥70%.^[34]^ The cut-off values on CTA should be 30% to detect all lesions ≥50% on DSA, by which the CTA has a similar high sensitivity and specificity for detecting the stenosis of large cerebral vessels.^[35]^ The stenosis is detected by 3D image of CTA, and then the level of stenosis is calculated by the 2D gray scale MPR images with window and level of 1000 and 500 HU (Fig. 2), respectively.^[36]^ AHA/American Stroke Association guidelines^[29]^ suggest that severe cerebrovascular stenosis is a contraindication for intensive treatment. The included patients are divided into groups according the location of stenosis, such as anterior circulation (internal carotid artery, anterior cerebral artery, middle cerebral artery, posterior cerebral artery) and posterior circulation (vertebral artery or basilar artery and others lesions). Raw contrast-enhanced CT images are analyzed by a technician and are checked by a senior technician to ensure the accuracy. The perhematoma region is defined as the region of 1 cm from the margin of hematoma, excluding subarachnoid and intraventricular space.^[37]^ The vessels are also removed from the region, which show as voxel with CBF >100 mL/100 g/min or 8 mL/100 g.^[38–40]^ The mean rCBF, Tmax, MTT, and rCBV of perhematoma region and contralateral homologous regions are calculated with removal of vessels. In addition, the mean rCBF, Tmax, MTT, and rCBV of hemisphere are also used to analyze the difference in 2 treatments. Hypoperfusion is defined as absolute CBF <18 mL/100 g/min, or delay of MTT and Tmax more than 2 seconds, and severe hypoperfusion is CBF <12 mL/100 g/min.^[41,42]^ Relative rCBF, Tmax, MTT, and rCBV are defined the ratio of parameters of perhematoma region to those of contralateral homologous regions (Fig. 2).

### 2.8 Secondary outcomes

The key secondary outcome is the clinical outcomes (death and disability) at 90-day. Disability is considered as modified Rankin score from 3 to 5 point. The other secondary outcomes include incidence of other vascular events, hospital stays, and health-related quality of life measured from the EQ-5D at 90 days.

### 2.9 Statistical analysis

The analysis of this study is based on the intention-to-treat principle. The continuous variable of primary outcome and second outcomes will be assessed by *t* test. The Chi-square test will be used to assess the categorical data of second outcomes. Log-rank test will be used to analyze the time-to-event type of outcomes. Subgroup analysis will be conducted by level of cerebrovascular stenosis and location of the cerebrovascular stenosis.

### 2.10 Data collection and management

Every patient included will have a unique study number. Allocation data will be collected by the special researcher and CRF will be filled by neurosurgeons with the study number. The follow-up data will be collected separately by another 2 trained investigators for the outcomes assessment. All data will be double inputted into a database and checked by Quality Monitoring Board (QMB) and the principal investigator, then be locked and sent to the statisticians.

### 2.11 Safety and data monitoring

Safety and data of this trial are monitored by the data and safety monitoring board (DSMB), which involves neurosurgeons, neurologists, radiologists, and biostatisticians. They meet each other and review the data including rate of adverse effects and dropout once half a year. Adverse effect is defined as any unexpected events of the included patients during the study. Severe adverse event is defined as death or a vegetative state. All of adverse effects are recorded in the CRF by times, events, and treatment measures. The DSMB will stop the trial and modify the protocol if one of the groups has a higher mortality or rate of adverse effects than the other with a significant difference of more than 3 SD.

### 2.12 Protocol amendment and dissemination

The protocol amendment must be approved by Biological and Medical Ethics Committee before implementation. The final findings will be disseminated to participants and their legal surrogate, scientific conferences, and publicly published in a peer-reviewed journal according to CONSORT guidelines.

### 2.13 Study organization and funding

The trial is conducted in the Department of Neurosurgery, West China hospital, Sichuan University, and supported by the Sichuan Province Science and Technology Support Project of the Science & Technology Department of Sichuan Province, China [grant number 2015SZ0051].

## 3 Conclusion

ATICHST has been signed as a parallel, prospective, randomized, assessor-blinded clinical trial of management of BP for sICH in patients with cerebrovascular stenosis, which is not demonstrated by now available study. The trial is aimed to assess the safety and effectiveness of intensive BP lowering for sICH cerebrovascular stenosis, which may result in reduction of peri-hematoma rCBF and ischemic stroke. A total of 80 patients will be involved in this trial. The relative reduction of peri-hematoma rCBF is analyzed as the key primary outcome. The absolute reduction of others cerebral perfusion indexes and the rate of ischemic stroke are regarded as other primary outcome. The secondary outcome includes clinical outcome at 30 and 90 days, complications, and hospital stays.
